# An Objective Walkability Index for Public Health and Planning in Peel Region, Ontario, Canada

**DOI:** 10.5888/pcd16.180469

**Published:** 2019-07-03

**Authors:** Maria Mukhtar, David Guillette, Natalie Lapos, Sandra Fitzpatrick, Ron Jaros

**Affiliations:** 1 Division of Chronic Disease and Injury Prevention, Region of Peel–Public Health, Ontario, Canada; 2 Division of Information Management, Region of Peel–Peel Data Centre, Ontario, Canada

**Figure:**
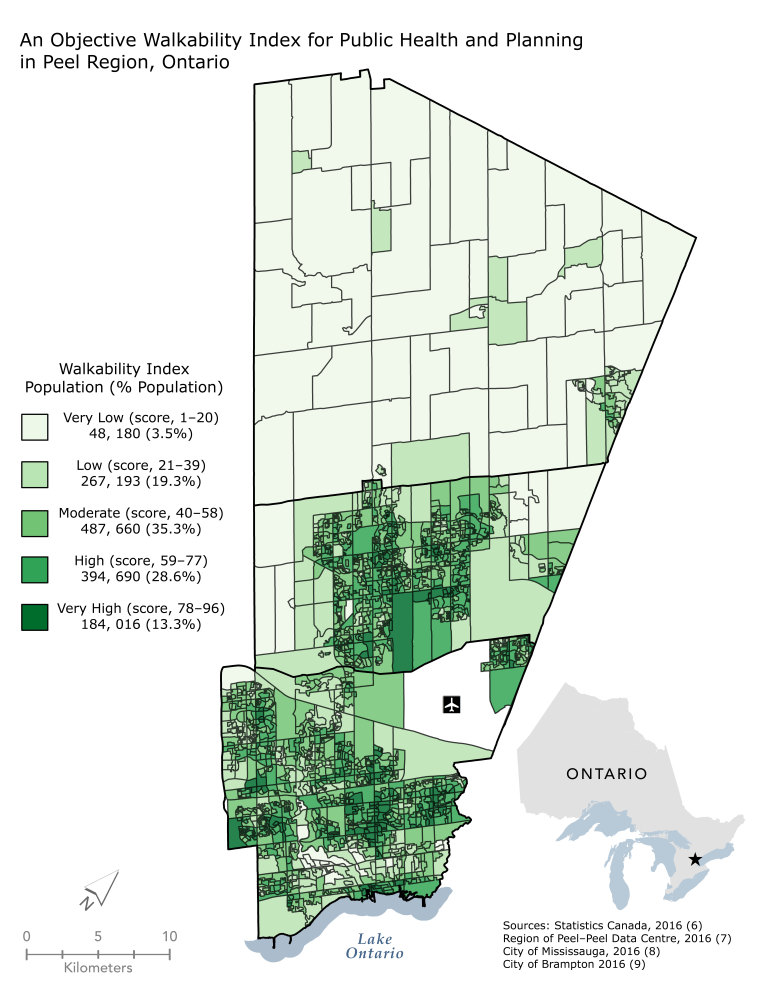
The Peel Walkability Composite Index is used as an evaluation tool and consists of 3 equally weighted components: access to retail and service outlets, access to schools and green spaces, and residential density and diversity. Understanding the capacity of the built environment to facilitate walking for utilitarian purposes (1) allows public health departments to advocate for strategic land use and infrastructure developments that promote an increase in population levels of physical activity.

## Background

During the past decade, autocentric suburban regions in Canada experienced tremendous growth. Autocentric built environments discourage active transportation and are linked to chronic disease risk factors (eg, low physical activity levels) (2). Peel Region is a large suburban municipality in Canada with a population of 1.38 million people and an average annual growth rate of 1.3%. To promote healthier communities, the Region of Peel–Public Health partnered with land-use planners on a public health intervention that incorporated policies in Peel’s Regional Official Plan (www.peelregion.ca/planning/officialplan), which requires a health assessment on development applications. Evaluation of this intervention relies on the Peel Walkability Composite Index (PWCI).

The PWCI is part of a larger initiative to produce indicators that measure and monitor built environment infrastructure throughout Peel. The PWCI includes indicators that measure built environment features influenced by the policy intervention. Collectively, the indicators operationalize the larger construct of neighborhood walkability and are thus composed into a single evaluation metric (ie, the PWCI).

Well-established walkability indices, including the Physical Activity in Localities and Community Environments (3) and the Neighborhood Quality of Life Study (4), empirically demonstrate the relationship between environmental attributes (ie, residential and retail density, street connectivity, and land-use mix) and physical activity outcomes. A lack of diversity in the attributes used to construct these indices is an acknowledged limitation (3,5). The PWCI was constructed by using a diverse range of objective indicators and was designed to ensure measurement repeatability.

## Data Sources and Map Logistics

The PWCI must be repeatable to capture differences in the index score over time. We created the PWCI in 2 stages: 1) we determined the measures to include in the index by using principal component analysis (PCA), and 2) we determined an appropriate weighting scheme to ensure measurement repeatability.

Using PCA on measures of density, diversity, and connectivity is a common approach to creating a walkability index. For the PWCI, we used PCA only to screen and select variables to construct the index. We completed PCA by using the following 14 indicators in SPSS software version 21.0.0.2 (IBM Corporation):

- residential density (Census 2016 [6])
- population density (Census 2016 [6])
- population-plus-employment density (Census 2016 [6] and Municipal Employment Surveys 2015–2016 [7–9])
- proximity of residents to frequent transit (Census 2016 [6] and General Transit Feed Specification 2016 [8,9])
- proximity of residents to green spaces (Census 2016 [6], Active Recreation, Parks, Trails, Peel Data Centre 2016 [7], Parks [8,9] and Conservation Areas [10,11])
- proximity of residents to food stores (Census 2016 [6] and Food Check Peel 2016 [7])
- proximity of residents to schools (Census 2016 [6] and Schools, Peel Data Centre 2016 [7])
- proximity of residents to community and retail services (Census 2016 [6], Municipal Employment Surveys 2015–2016 [7–9], Food Check Peel 2016 [7] and Child Care, Land Marks, Peel Data Centre 2016 [7])
- diversity of land use (Parcel Based Land Use 2016 [7])
- diversity of housing stock (Census 2016 [6])
- intersection density (Single-Line Street Network, Peel Data Centre, 2016 [7])
- percentage of sidewalks with tree canopy (Peel Land Cover, Peel Data Centre 2016 [7] and Sidewalks 2016 [7–9])
- proximity of residents to bicycle networks (Census 2016 [6] and Trails, Peel Data Centre 2016 [7])
- percentage of local roads with speeds below 40 km/hour (Single-Line Street Network, Peel Data Centre 2016 [7])

Indicators had high face validity and were constructed at the level of the Canadian Census dissemination area. We calculated proximity indicators by using 400-m, 800-m, or 1,600-m network distances from points of interest to residential parcels to account for population weighting within dissemination areas. We standardized indicator values by *z* scores before inclusion in the PCA.

Because of multicollinearity (bivariate correlation scores >0.8), inadequate measures of sampling adequacy (values <0.5 from anti-image correlation matrix), and high levels of nonredundant residuals (>0.05), we removed 6 of the 14 indicators from the PCA: population density, population-plus-employment density, intersection density, percentage of sidewalk with tree canopy, proximity of residents to bicycle networks, and percentage of local roads with speeds below 40 km/hour. We extracted 3 components with eigenvalues greater than 0.95; these components accounted for 62.4% of the total variance. The retained 8 indicators loaded on 3 components: access to retail and service outlets (proximity of residents to grocery stores, +0.85; proximity of residents to community and retail services, +0.85; diversity of land use, +0.57); access to schools and green spaces (proximity of residents to green spaces, +0.80; proximity of residents to schools, +0.74); and residential density and diversity (residential density, +0.86; diversity of housing stock, +0.73, proximity of residents to frequent transit, +0.43).

We constructed the PWCI by averaging the sum of the normalized scores for the standardized indicators that loaded on the extracted principal components for each dissemination area. We normalized the retained 8 indicators (on a scale of 0 to 100) and averaged them by using equal weighting to create the composite index. This process resulted in dissemination area PWCI scores ranging from 1 to 96. These scores provide a 2016 benchmark walkability score. Using equal weighting ensures that component loading values will not influence the capacity of the PWCI to monitor changes in scores over time. We divided the composite index into 5 classes in equal intervals of walkability, from very low (score of 1–20) to very high (score of 78–96). We mapped these classes to illustrate the spatial distribution of walkability in Peel.

## Highlights

Many residents of Peel (41.9%) live in areas classified as highly or very highly walkable. These areas are in the downtown cores of cities that have zoning bylaws that encourage higher density and greater mix of land use. Approximately one-third of residents (35.3%) live in a moderately walkable area. These areas are in the inner suburbs, close to city cores, and benefit from proximity to schools and green space. Almost a quarter of residents (22.8%) live in areas with very low or low walkability, along suburban edges. A cluster of areas with very low walkability in the southwest is due to pedestrian barriers, including a highway and the Credit River. Planning policies in these areas encourage very low-density development, contributing to minimal walkable destinations.

## Action

The indicator data for the PWCI will be rerun every 5 years, in sequence with the Canadian Census, to monitor changes in the spatial distribution of walkability in Peel. The PWCI is an evidence-informed tool that local elected officials, planners, and public health departments can use to evaluate health-promoting built environment policies and inform future land-use policies. Understanding the spatial distribution of walkable built environments promotes strategic investments in infrastructure that are aimed at increasing levels of physical activity among adults (1).
